# Artificial Urinary Sphincter-Sparing Vulvoplasty: A Case Report

**DOI:** 10.7759/cureus.104197

**Published:** 2026-02-24

**Authors:** Steven Zeng, Brendan Wallace, Armina Azizi, Tyler Garman, Benjamin Rail, Andrew Cohen, Fan Liang

**Affiliations:** 1 Plastic Surgery, Johns Hopkins Health System, Baltimore, USA; 2 Urology, Johns Hopkins Health System, Baltimore, USA; 3 Center for Transgender and Gender Expansive Health, Johns Hopkins University, Baltimore, USA

**Keywords:** artificial urinary sphincter, gender health, surgical case reports, transgender surgery, vaginoplasty

## Abstract

Artificial urinary sphincters (AUSs) are a standard treatment for post-prostatectomy incontinence. The risk of AUS erosion after future urethral surgery could be seen as a contraindication to vulvoplasty in transgender patients. However, we describe a novel AUS capsule-sparing vulvoplasty technique that minimizes this risk. A 60-year-old transgender female with a history of bilateral orchiectomy, urethral sling excision, and AUS placement for post-prostatectomy incontinence sought vulvoplasty for gender-affirming care and wished to preserve her AUS. Risks, including erosion, infection, and AUS malfunction due to limited tissue coverage, were discussed. A combined plastic surgery and urology team performed the procedure. The AUS was deactivated, and a suprapubic tube was placed. Scrotal skin was excised, leaving the Dartos fascia over the pump intact. Dissection proceeded to the bulbospongiosus muscle, sparing the AUS capsule and tubing. A non-standard urethrectomy preserved urethral tissue, with the neomeatus created 2 cm distal to the capsule for protection. The AUS pump was repositioned beneath the left neolabia majora, constructed from perineal and scrotal flaps. After skin closure, the AUS was tested and reactivated. At follow-up, the patient was satisfied with vulvoplasty and AUS function. We present a novel AUS-sparing vulvoplasty technique for gender-affirming surgery (GAS) that preserves continence support in a complex post-prostatectomy patient.

## Introduction

Gender-affirming surgery (GAS), including vulvoplasty, can significantly improve mental health, quality of life, and overall well-being for transgender patients. The World Professional Association for Transgender Health (WPATH) emphasizes that access to these surgeries can be life-saving and can reduce the risk of suicide and self-harm [1]. 

Vulvoplasty, also known as zero-depth or minimal-depth vaginoplasty, involves the construction of a vulva without creating a vaginal canal. Creation of a vaginal canal may be undesirable due to patient concern about complications, inability, or unwillingness to commit to post-operative dilation, absence of dysphoria related to vaginal depth, or prior surgical history that complicates full-depth vaginoplasty.

AUS is a common surgical treatment for post-prostatectomy incontinence. The device consists of a urethral cuff, a fluid reservoir placed in the retropubic space, and a hemi-scrotal pump. Subsequent surgical manipulation of the urethra after AUS placement carries a high risk of device erosion (8%) [2]. In cases of erosion, up to 33% of patients may develop urethral sphincter damage, potentially resulting in severe, long-term incontinence [3]. Therefore, in patients with an existing AUS who are seeking vulvoplasty, multidisciplinary planning is essential to determine the surgical approach, appropriate urethral length, and whether device relocation is necessary. The following case is the first description of an AUS-sparing vulvoplasty technique and is presented in accordance with the CARE guidelines.

This article was previously presented as a meeting abstract at the Genitourinary Reconstructive Surgery Meeting in September 2025.

## Case presentation

We present a novel modification to vulvoplasty for patients with an existing AUS. A 60-year-old transgender female with a past surgical history of robot-assisted radical prostatectomy for prostate cancer, simultaneous bilateral orchiectomy, urethral sling excision, and AUS placement for persistent post-prostatectomy incontinence sought a vulvoplasty for gender-affirming care with preservation of AUS. The risks of device erosion and infection were discussed, as well as potential malfunction secondary to decreased device tissue coverage. The surgery was performed by a combined plastic surgery and urology team. The patient has consented to the disclosure of this case report, and her signed consent has been included in the journal submission process.

**Figure 1:**
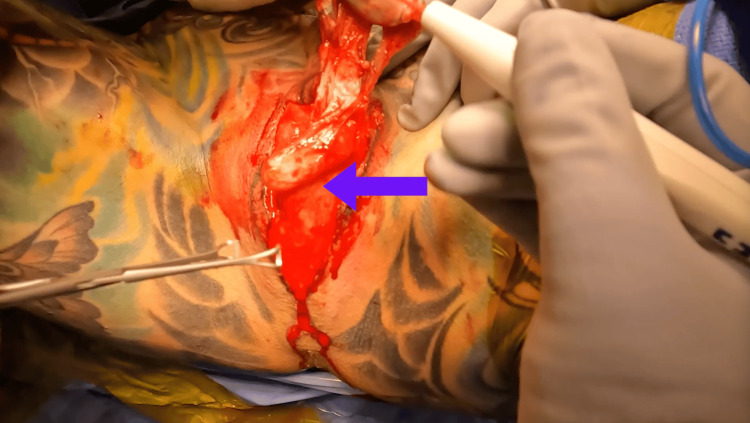
Identification and retraction of the AUS device during skin excision shown with arrow. AUS: artificial urinary sphincter

To begin, the AUS was deactivated, and a suprapubic tube was placed under direct vision. The scrotal skin was excised, but the Dartos fascia overlying the pump in the left hemi-scrotum was left intact (Figure 1). 

**Figure 2:**
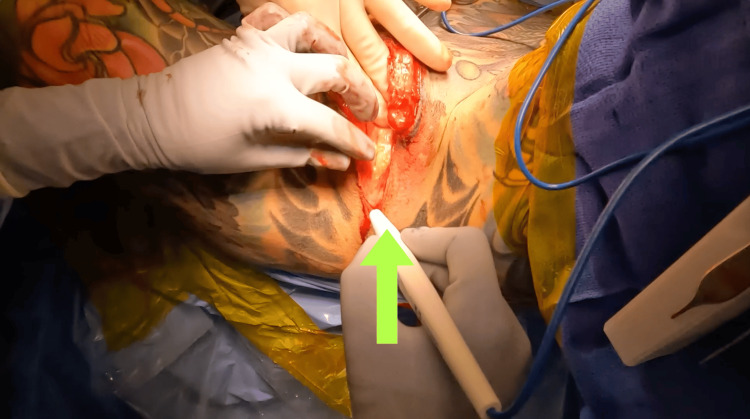
Identification and sparing of the AUS cuff and capsule during bulbospongiosus dissection, shown with an arrow. AUS: artificial urinary sphincter

The standard dissection of the bulbospongiosus muscle was completed more distally than normal to spare the AUS cuff capsule and its associated tubing (Figure 2).

**Figure 3:**
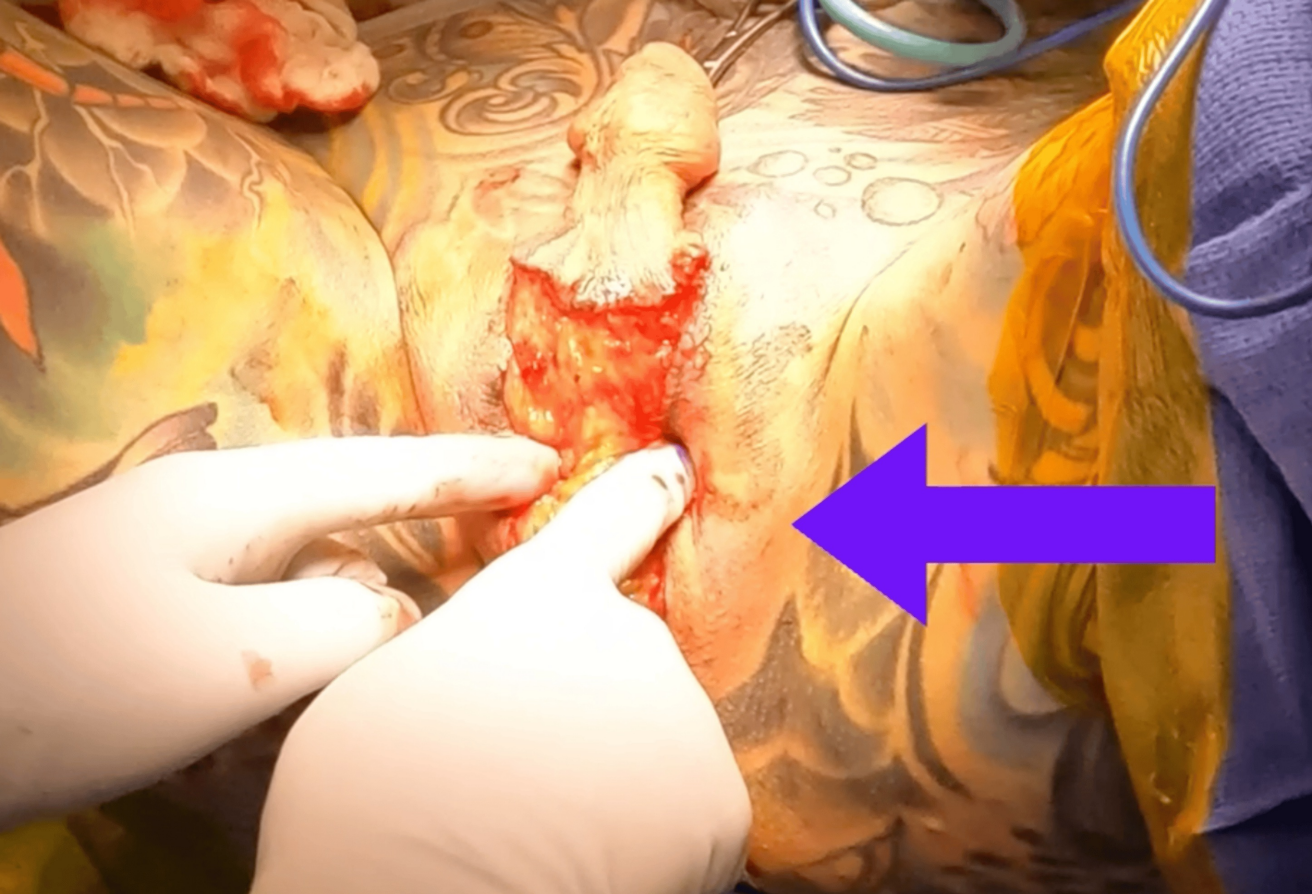
Manipulation of the AUS under the left neolabia, shown with an arrow. AUS: artificlal urinary sphincter

Urethrectomy was also performed in a non-standard manner, with the creation of the neomeatus 2 cm distal to the cuff capsule to preserve urethral tissue and provide coverage for the cuff. The AUS pump was manipulated to lie under the left neolabia majora, which was composed of perineal and scrotal flaps (Figure 3). 

Antibiotic irrigation was used throughout the case. Following skin closure, the AUS was tested for ease of use and reactivated. The device remained activated during the post-operative period. All portions of the case are summarized in Video 1.

**Video 1:** Intraoperative recording highlighting the key portions of an AUS-sparing vulvoplasty. AUS: artifical urinary sphincter

The patient was discharged on post-operative day four with a course notable for a two-day readmission on post-operative day six for *Serratia* bacteremia. The patient’s suprapubic tube was removed one month after surgery with satisfactory labial positioning of the AUS pump. At nine months, the AUS was functioning without issue, and the patient reported no issues with urinary spraying. Her urinary stream was inferiorly directed with her hips adducted. She is also able to achieve orgasm and is satisfied with her aesthetic result.

## Discussion

Vulvoplasty alters the form and function of the native penile anatomy, with the goal of creating an aesthetic neovagina while preserving urinary function. Key goals include well-defined, three-dimensional labia minora that frame the introitus, sufficient clitoral hooding, a closed-appearing introitus at rest, and prominent, symmetric labia majora [1-6]. The authors present a novel approach to AUS-sparing vulvoplasty with modifications to the urethrectomy, urethroplasty, and construction of the labia majora.

**Figure 4:**
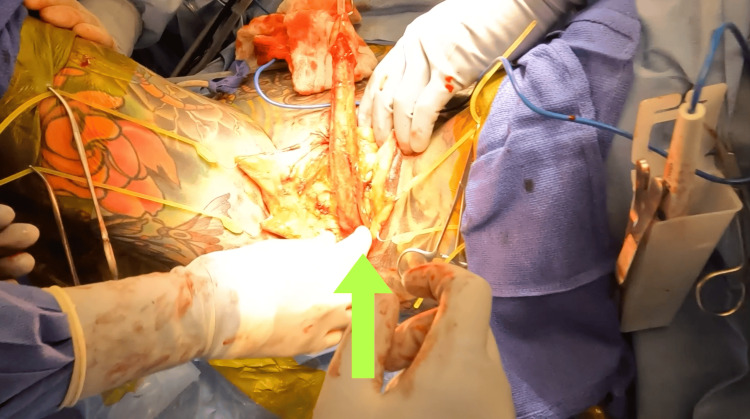
Determination of distal urethrectomy location, shown with an arrow.

Urinary complaints have been reported in up to 30% of patients following vaginoplasty [4,5]. One cause of patient distress is a diverted or poorly directed urinary stream [7]. The typical male urethra follows an S-shaped curve defined by the penile suspensory ligament, creating an anterior curvature in the penis and urethra [8]. During a standard urethrectomy, the urethra is transected at the level of the pubic bone and split down the ventral midline proximally, resulting in an inferiorly directed urethra and loss of its native anterior curvature [9]. In this case, the urethra was truncated more distally to avoid the AUS and its associated components (Figure 4).

While this preserved urethral segment provided additional soft tissue coverage for the AUS, it also maintained some of the urethra’s natural anterior curvature, risking an anteriorly directed urinary stream postoperatively. To address this, the posterior wall of the urethral meatus was everted during the urethroplasty to create an inferiorly facing neomeatus.

The added urethral length also introduced anatomical challenges. Distal to the AUS, the bulbar urethra is surrounded by corpus spongiosum, which can engorge during arousal and, if left intact, can lead to visible bulging, obstructed voiding, difficulty with intromission, and patient distress [9]. To prevent this, the spongy tissue of the ventral bulb was aggressively excavated and excised, with care taken not to injure the soft tissue capsule over the AUS cuff.

Finally, special care was taken to achieve symmetry in the labia majora, particularly on the side contralateral to the AUS. Asymmetry is a common reason for revision after GAS, with labiaplasty frequently performed to address contour concerns. [4] To reduce the likelihood of future revision, excess subcutaneous tissue was preserved to create a deliberately full-appearing labia majora, an approach that may provide a sustained aesthetic result. Furthermore, to define the sulcus separating the labia majora and minora, the author typically places horizontal mattress sutures. However, to avoid damage to the AUS, these sutures were omitted on the device-side, resulting in a less defined intra-labial sulcus and sacrificing symmetry for AUS preservation.

## Conclusions

A prior history of AUS placement should not be regarded as a contraindication to gender-affirming vulvoplasty. This case demonstrates that with careful preoperative planning, multidisciplinary collaboration, and targeted intraoperative modifications, including AUS capsule preservation, strategic urethral transection, and tailored labial reconstruction, patients can achieve both functional and aesthetic outcomes without compromising urinary continence.

Our novel AUS-sparing vulvoplasty technique offers a viable option for transgender patients with complex urologic histories, expanding surgical candidacy for a population that often faces limited reconstructive options. Further studies are warranted to assess long-term outcomes, complication rates, and patient satisfaction following AUS-sparing vulvoplasty. As the field of transgender surgery continues to evolve, expanding the armamentarium of techniques that accommodate prior surgical history will be essential in providing equitable, individualized care.
